# Invited Commentary: Reducing the Risk of Anastomotic Leak After Rectal Cancer Resection

**DOI:** 10.1002/wjs.70276

**Published:** 2026-02-17

**Authors:** Henry Richard Edward Drysdale, David Allan Watters

**Affiliations:** ^1^ Department of Surgery University Hospital Geelong Barwon Health Geelong Victoria Australia; ^2^ Deakin University School of Medicine and Health Sciences Waurn Ponds Victoria Australia

**Keywords:** anastomotic leak, colorectal surgery, elective surgery, surgical outcomes, unplanned readmission

## 1 Invited Commentary

Anastomotic leak (AL) remains a feared complication for the Colorectal Surgeon and their patients [1]. It is associated with significant patient morbidity, poor long‐term functional outcomes, and potential compromise of oncological outcomes [2, 3, 4]. Compared to other colorectal resections, low anterior resections (LAR) are associated with higher rates of AL (up to 25% [1]) with increased risk of long‐term complications [5, 6]. Therefore, it is at the forefront of every colorectal surgeon's mind as to how to decrease the risk of AL during LAR.

An enhanced recovery after surgery (ERAS) program has demonstrated improved outcomes for patients undergoing colorectal resections [7]. Furthermore, patients undergoing anterior resection under an ERAS protocol have been shown to have lower rates of AL. A Swedish cohort of 1900 patients reported 155 ALs (8.2%), with a higher rate of leaks associated with male sex, obesity, peritoneal contamination, duration of surgery, and age. Preoperative patient education was shown to reduce AL, whereas there was no difference in ERAS bundle compliance between those with and without leaks [8].

Although the exact content of an ERAS bundle varies between institutions and service providers, all bundles cover a range of preoperative, intraoperative, and postoperative items [9]. ERAS protocols do not usually include the stipulation of more technical aspects of surgery, such as reinforcement of an anastomotic staple line or the use of indocyanine green to evaluate anastomotic perfusion. Therefore, the impact of a technically‐focused ERAS bundle on anastomotic leak is unknown.

In this issue of the journal, Tamura et al. report a remarkably low leak rate (2.5%) in a small series of 81 patients who underwent low (LAR) and ultralow anterior resection (ULAR) for rectal cancer. Their institution had already introduced a standardized ERAS protocol, which was consistent with many features of published ERAS guidelines [9]. In response to an anastomotic leak rate of 14.7%, they added to the ERAS package a number of “rectal surgery‐specific” elements in an attempt to reduce anastomotic leakage. These included preoperative oral antibiotics (70/81 and 86.4%), robotic assisted surgery (72/81 and 88.9%), indocyanine green assessment of perfusion (80/81 and 98.8%), transanal drainage tube insertion (66/81 and 84.1%), anastomotic reinforcement when performing ULAR (29/39 and 74.4%), and, for ULAR, the use of a diverting stoma (33/81 and 40.7%) [10].

The introduction of a rectal surgery‐specific surgical care bundle had a significant impact on decreasing anastomotic leak (14.7% vs. 2.5% and *p* < 0.01), including after propensity score matching (18.0% vs. 1.3% and *p* < 0.01). There were no unplanned returns to theater whereas Clavien–Dindo 3 complications and surgical site infections were significantly reduced [10]. When compared with the historical cohort, the later cohort with a lower leak rate had less diabetics, less lateral lymph node dissections, a shorter operating duration, and less blood loss but similar proportions of patients who were male (58%), obese (22%), smokers (20%), or had undergone neoadjuvant therapy (18%).

Despite the differences between their cohorts, the remarkably low leak rates for LAR and ULAR (2.5%) in the latter cohort are impressive and the various elements implemented deserve consideration. What remains the largest challenge in analyzing Tamura et al.‘s impressive results is breaking down the individual components of their bundle and their potential impact on the anastomotic leak rate. As with the analysis of any surgical care bundle, trying to work out which components are responsible for influencing the outcome is usually not possible. Compounding this uncertainty is the variability in implantation of the specific aspects of the rectal cancer‐specific bundle in Tamura et al.‘s cohort. With the final decision for implementation left to the treating surgeon, each aspect of the bundle was implemented in 40.7%–98.8% of cases, leading to significant heterogeneity of care. What can be derived from the data is that the surgical care bundle created a framework for decision making that optimized individual surgeon decision making, ultimately leading to improved patient outcomes.

The applicability of these results to the broader population of rectal cancer patients must be made with caution. Importantly, open surgery was excluded from the analysis which may have led to the exclusion of more technically challenging cases that were chosen to be done open due to either patient and/or disease characteristics. Additionally, there were relatively low rates of neoadjuvant treatment with overall only 53 of 306 (17.3%) of patients receiving neoadjuvant treatment, with no significant difference between patients pre and postbundle (*p* = 0.73). These rates are significantly lower than other countries, where reported rates of neoadjuvant therapy are up to 90% [11].

Decreasing the rate of anastomotic leak is only one aspect of a surgeon's role when performing a colonic or rectal anastomosis. Despite our multifaceted efforts, the occurrence of an anastomotic leak is not always avoidable. Early detection of an anastomotic leak is associated with improved patient’s outcomes [12]. Close clinical observation for failure to progress or clinical deterioration is essential. Biochemical monitoring, including with C‐reactive protein (CRP), remains a well‐proven adjunct [13]. Recent data suggest that the CRP threshold in robotic colorectal resections are lower than reported following open and laparoscopic surgery [14].

The management of all anastomotic leaks requires careful consideration; however, those that occur below the peritoneal reflection require even more nuanced management. Leaks below the peritoneal reflection can lead to significant mortality and morbidity including the inability to restore gastrointestinal continuality. Management options include antibiotics, transanal drainage (including VAC therapy), transanal closure, percutaneous drainage, or return to theater for a defunctioning or end colostomy [15]. The majority of patients in Tamura et al.‘s paper that underwent an anastomotic leak were managed nonoperatively (reoperation rate 3% overall), highlighting the varying options available for management of the anastomotic leak below the peritoneal reflection.

Although Tamura et al.‘s results highlight the potential benefits of an ERAS‐based surgical care bundle on decreasing anastomotic leak, the study remains a small series (81 patients) of retrospectively analyzed low and ultralow anterior resections. Further research in the form of multicenter prospective studies are required to determine the full‐impact of these ERAS‐based and additional “rectal surgery specific” measures.

## Author Contributions

**Henry Richard Edward Drysdale:** writing – original draft, methodology, investigation, visualization. **David Allan Watters:** conceptualization, writing – review and editing, validation, visualization, project administration.

## Funding

The authors have nothing to report.

## Conflicts of Interest

The authors declare no conflicts of interest.

## Data Availability

The data in this paper are available from the cited papers in the reference list.
